# Effect of surface state hybridization on current-induced spin-orbit torque in thin topological insulator films

**DOI:** 10.1038/s41598-017-00911-4

**Published:** 2017-04-11

**Authors:** Cong Son Ho, Yi Wang, Zhou Bin Siu, Seng Ghee Tan, Mansoor B. A. Jalil, Hyunsoo Yang

**Affiliations:** 1 Department of Electrical and Computer Engineering, National University of Singapore, 4 Engineering Drive 3, Singapore, 117576 Singapore; 2 Data Storage Institute, Agency for Science, Technology and Research (A*STAR), 2 Fusionopolis Way, 08-01 Innovis, Singapore

## Abstract

We investigate the thickness optimization for maximum current-induced spin-orbit torque (SOT) generated by topological surface states (TSS’s) in a bilayer system comprising of a ferromagnetic layer coupled to a thin topological insulator (TI) film. We show that by reducing the TI thickness, two competing effects on the SOT are induced: (i) the torque strength is stronger as the bulk contribution is decreased; (ii) on the other hand, the torque strength becomes suppressed due to increasing hybridization of the surface states. The latter is attributed to the opposite helicities of the coupled TSS’s. We theoretically model the interplay of these two effects and derive the optimal TI thickness to maximize the spin torque, which is estimated to be about 3–5 nm for typical Bi_2_Se_3_ films.

## Introduction

Spin torque is one of the most actively researched topics in spintronics. In spin-orbit coupling (SOC) systems, the spin-orbit torque (SOT) comprises of two types: field-like torque^1–6^ and damping-like torque^7, 8^. The SOT strength scales with the SOC strength, therefore one can obtain a large SOT signal in strong SOC systems, such as heavy metal/ferromagnet (Pt/Co)^9^ and topological insulators (TI)^10–12^.

Topological insulators possess strong SOC which translates to a large SOT effect. Moreover, the spin-locked topological surface states of TI are robust under time-reversal symmetric impurity scattering. These factors enable TI to be one of the most promising candidates for SOT devices. When a current is passed onto the TI surface (interface), a non-equilibrium spin density will be induced on that surface^10^, which is directed in-plane and perpendicular to the applied current as a result of the spin-momentum locking of the surface states^10^, similar to the Rashba-Edelstein effect^13^. Thus spin accumulation is proportional to the charge current flowing on the TI surface^1, 3, 10^. In addition, an out-of-plane spin density can be generated due to the Berry curvature of the spin texture in the momentum space^8, 14, 15^, and due to the presence of the hexagonal-warping effect^16^. The two components, in-plane and out-of-plane spin density, are responsible for inducing an out-of-plane and in-plane torques, respectively.

In the study of current-induced SOT, spin torque efficiency (ratio of torque strength and current density in the TI - ${\mathscr{T}}/{j}_{{\rm{TI}}}$, which is equivalent to spin Hall angle *j*_*s*_/*j*_TI_) is a crucial quantity. In 3D TI’s, the surface state has a finite thickness and usually extends into the bulk material, up to 1 nm from the TI-vacuum interface^11, 17^, and the current is supposed to flow entirely in the surface channel in ideal TI. However, in practical 3D TI’s, the bulk is not totally insulating^18^, and thus the current in the TI can flow both in the surface and bulk channels. Since the bulk states do not exhibit any spin-momentum locking, TI with a large bulk conductance will induce a smaller torque on an adjacent ferromagnetic (FM) layer. Therefore, it is obvious that one has to reduce the TI thickness to reduce the relative contribution of the bulk^19–21^, and enhance the torque efficiency. But how far can the thickness of a TI film be reduced?

Normally, a 3D TI film comprises of two surfaces (top and bottom surfaces, for example). In a typically thick TI film, the two surfaces are uncorrelated, i.e., the transport on one surface will not affect the other. However, if the film is made very thin, *i*.*e*., its thickness is comparable to the decay length of surface states, the two surface states become hybridized^17, 22–24^. This hybridization is quantified by a tunneling coupling constant (Δ), which is thus a function of the TI thickness. Generally, Δ is larger as the thickness is reduced^17, 22, 23^. The effect of the surfaces hybridization on spin torque is as follows. When a charge current is passed onto the top surface in the *x*-direction, it can leak to the bottom surface through the tunneling effect^25^. While the current on the top surface induces a spin accumulation in the *y*-direction, its bottom counterpart induces a spin accumulation in the opposite, *i*.*e*., –*y*-direction, since the two surface states have opposite helicities. As a result, the total spin accumulation is reduced with the hybridization effect. Thus, the spin torque is also reduced as the TI thickness is reduced. This dependence will be formulated subsequently in this paper.

From the above, we may surmise that the reduction in the TI thickness has two competing effects on the spin torque: (i) the torque strength is stronger as the bulk contribution is decreased; (ii) on the other hand, the torque strength becomes suppressed due to increasing hybridization of the surface states. Therefore, there is an optimal value of TI thickness which would maximize the torque efficiency. Thus, the aim of this study is to investigate the spin torque in TI film in the presence of the hybridization effect. We will formulate the relation between spin torque and TI thickness, from which we derive the optimal value of the thickness to maximize the torque.

## Results

### Spin torque in thick TI films

**Figure 1:**
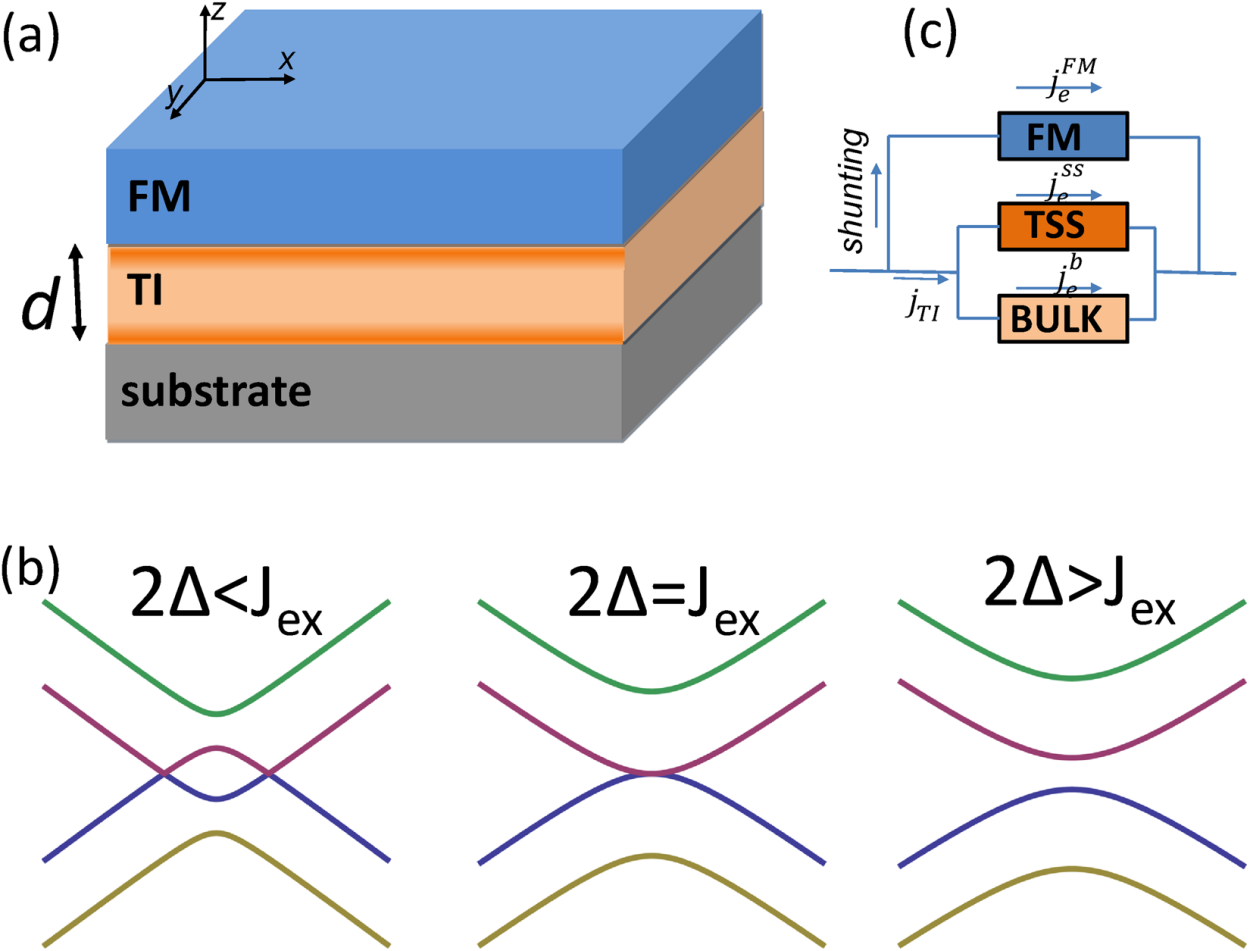
(**a**) Schematic diagram of bilayer ferromagnet/topological insulator. The TI film is of the thickness *d*. (**b**) Schematic energy ${\mathcal E}$ versus *k*_*y*_ subbands near the Dirac points (*k*_*x*_ = 0) for various values of the tunneling element Δ. (**c**) Schematic diagram of the current distribution in the bilayer with *j*_*TI*_ being the current in the TI film, and ${j}_{e}^{FM}$ being the shunting current through the FM layer. In the TI film, the current can flow in the surface state channel $({j}_{e}^{ss})$ and bulk channel $({j}_{e}^{b})$, respectively.

We will first formulate the spin-torque induced by charge current in a coupled FM/TI bilayer system in the absence of inter-surface hybridization. In a thick TI film, only the topological surface state coupled to the magnetic layer is active (the top TSS in Fig. 1), whereas the other has no effect and thus can be ignored (the bottom TSS). The system is described the by following Hamiltonian:

1 $$ {\mathcal H} ={ {\mathcal H} }_{{\rm{TI}}}+{J}_{{\rm{ex}}}{\bf{m}}\cdot \hat{\sigma },$$

where the first term is the Dirac Hamiltonian of the active topological surface state ${ {\mathcal H} }_{{\rm{TI}}}=\hslash {v}_{{\rm{F}}}({\bf{k}}\times \hat{z})\cdot \hat{\sigma }$ with *v*_F_ being the Fermi velocity, $\hat{\sigma }$ is the vector of the Pauli matrices. The last term is the *s*-*d* exchange interaction between itinerant electrons and magnetization with *J*_ex_ being the exchange constant, and **m** is unit vector of the magnetization which is assumed to be in-plane. We can rewrite Eq. (1) to express electron spin in an effective magnetic field as ${\mathcal H} ={\bf{b}}\cdot \hat{\sigma }$, where ${\bf{b}}=\hslash {v}_{{\rm{F}}}({\bf{k}}\times \hat{z})+{J}_{{\rm{ex}}}{\bf{m}}$. The eigenenergies are found as

2 $${ {\mathcal E} }_{s}({\bf{k}})=s\sqrt{{(\hslash {v}_{{\rm{F}}}k)}^{2}+{({J}_{{\rm{ex}}})}^{2}+2\hslash {v}_{{\rm{F}}}{J}_{{\rm{ex}}}{\bf{m}}\cdot ({\bf{k}}\times \hat{z})}$$

where *s* = ±1 which is for majority and minority electron, respectively, and we have used the fact that |**m**| = 1. For a given value of momentum **k**, Eq. (2) can be considered as the energy of the magnetic system. Thus, the torque field acting on the magnetization can be found by taking the functional derivative of ${ {\mathcal E} }_{s}({\bf{k}})$, *i*.*e*.^1,2^,

3 $${{\bf{H}}}^{{\rm{eff}}}=-\frac{n}{{\mu }_{0}{M}_{s}}\frac{\delta  {\mathcal E} }{\delta {\bf{m}}}.$$

In the above, *n* is the charge density, and *M*_*s*_ is the saturated magnetization. Let us first consider the contribution from the lower band. In this case, from Eqs (2) and (3), the torque field is

4 $${{\bf{H}}}^{{\rm{eff}}}=\frac{n\hslash {v}_{{\rm{F}}}}{{\mu }_{0}{M}_{s}}({\bf{k}}\times \hat{z}).$$

The upper band will gives rise to an opposite torque field. To obtain the above, we have assumed the strong exchange limit, i.e., ${J}_{{\rm{ex}}}\gg \hslash {v}_{{\rm{F}}}{k}_{{\rm{F}}}$, $|b|\approx {J}_{{\rm{ex}}}$, and the effective field is taken to the first order in *ħv*_F_ *k*/*J*_ex_. The net torque field is found by summing over all momentum space. In the absence of applied current, the expectation value of **k** would vanish and so would the effective field. However, in the presence of some charge current ${{\bf{j}}}_{e}^{ss}$ that flows on the surface, the average **k** is finite and can be found as following. The charge current is expressed as ${{\bf{j}}}_{e}^{ss}=ne\langle {\bf{v}}\rangle$, where *n* is the sheet carrier density, and ${\bf{v}}=\frac{\partial H}{\hslash \partial k}={v}_{{\rm{F}}}(\hat{z}\times \hat{\sigma })$ is the velocity. In the adiabatic limit, the electron spin is mostly aligned along the effective field as $\langle \hat{\sigma }\rangle ={\bf{b}}/|b|$, from which the charge current is ${{\bf{j}}}_{e}^{ss}=ne\frac{\hslash {v}_{F}^{2}}{{J}_{{\rm{ex}}}}\langle {\bf{k}}\rangle +ne{v}_{{\rm{F}}}(\hat{z}\times {\bf{m}})$. Rearranging the equation, we have

5 $$\langle {\bf{k}}\rangle =\frac{{J}_{{\rm{ex}}}}{ne\hslash {v}_{F}^{2}}{{\bf{j}}}_{e}^{ss}-\frac{{J}_{{\rm{ex}}}}{\hslash {v}_{{\rm{F}}}}(\hat{z}\times {\bf{m}}).$$

Substituting Eq. (5) into Eq. (4), we obtain the current-induced torque field,

6 $${{\bf{H}}}^{{\rm{eff}}}={\eta }_{0}({{\bf{j}}}_{e}^{ss}\times \hat{z}),$$

where ${\eta }_{0}=\frac{{J}_{{\rm{ex}}}}{e{v}_{{\rm{F}}}{\mu }_{0}{M}_{s}}$, and we have ignored the component that is parallel to **m**, as this does not induce torque on the magnetization itself. The field in Eq. (6) is in-plane and it induces an out-of-plane torque on the magnetization as ${\mathscr{T}}={\bf{m}}\times {{\bf{H}}}^{{\rm{eff}}}$. Explicitly,

7 $${\mathscr{T}}={\eta }_{0}{\bf{m}}\times ({{\bf{j}}}_{e}^{ss}\times \hat{z}).$$

In practice, an applied current ${j}_{e}^{0}$ in the system is composed of three different channels: (i) surface current ${j}_{e}^{ss}$, (ii) bulk current ${j}_{e}^{b}$, and (iii) shunting current through the FM layer ${j}_{e}^{{\rm{FM}}}$, see Fig. 1(c). Therefore, from the total applied charge current ${j}_{e}^{0}$, only ${j}_{e}^{ss}$ would generate a torque field and spin torque, following Eqs (6) and (7). To obtain the relation between the surface current and the net current, we assume that the FM and TI layers have thicknesses *t* and *d*, and conductances *G*_FM_ and *G*_TI_, respectively. The surface current is then given by

8 $${j}_{e}^{ss}(d)={j}_{e}^{0}\frac{{G}_{{\rm{TI}}}^{ss}}{{G}_{{\rm{TI}}}+{G}_{{\rm{FM}}}}={j}_{TI}\frac{{G}_{{\rm{TI}}}^{ss}}{{G}_{{\rm{TI}}}}.$$

In the above, *j*_*TI*_ is the current flowing in the TI film, ${G}_{{\rm{TI}}}^{ss}$ is the conductance of TI surface, which is assumed to be unchanged as the thickness of the TI layer *d* is changed. Meanwhile, the total conductance of the TI layer *G*_TI_ increases with *d* ^18^, reflecting the increased contribution of the bulk channel. Therefore, the spin torque and torque field would generally be enhanced as *d* is reduced. In practice, TI films can be made very thin, *i*.*e*., down to 2 nm^18^, and therefore one may induce large spin torque in such systems. However, as the TI film thickness is reduced to below some critical value, the property of the surface state will be changed, *i*.*e*., an energy gap is opened and the Dirac cone would disappear, as a result of the surface hybridization^17, 21, 22, 24^. As a consequence, all spin transport effects arising from the topological surface states will be modified accordingly. The effect of surface hybridization will be considered in the following section.

### Spin torque with hybridization effect

#### Effect of surface hybridization

In this section, we will formulate the spin-orbit torque in a thin TI film with hybridization effect. In this case, both surface states (of top and bottom surfaces) are taken into account. We assume that the magnetic layer is only in contact with the top TI surface with the exchange coupling *J*_ex_. The itinerant electron on the bottom surface may also couple to the FM, but with a weaker exchange coupling, which is then ignored for simplicity. In addition, we also disregard the asymmetry between the two interfaces, *i*.*e*., FM/TI and TI/substrate interfaces.

The Hamiltonian of the system is then given by^24^

9 $$ {\mathcal H} =[\begin{matrix}{ {\mathcal H} }_{{\rm{TI}}}+{J}_{{\rm{ex}}}{\bf{m}}\cdot \hat{\sigma } & {\rm{\Delta }}{I}_{2}\\ {\rm{\Delta }}{I}_{2} & -{ {\mathcal H} }_{{\rm{TI}}}\end{matrix}].$$

In the above, Δ represents the tunneling element (hybridization) between top and bottom surfaces, *I*_2_ is the (2 × 2) unit matrix. The value of Δ has an inverse relation with the thickness of the TI film, e.g., Δ = 0.05 eV for *d* = 5 nm film, but increases to 0.25 eV for *d* = 2 nm^20^. In the range of small thickness, the tunneling element can be approximated as^17, 22^

10 $${\rm{\Delta }}\approx \frac{{B}_{1}{\pi }^{2}}{{d}^{2}},$$

where *B*_1_ is a material-dependent parameter^21^. Note that the Fermi velocity is also thickness-dependent. However, the variation can be neglected as its values in the thick and thin film limits only differ by the order of 0.01^22^.

From the Hamiltonian in Eq. 9, the eigenenergies are given by

11 $${ {\mathcal E} }_{s\tau }=s\sqrt{U+\tau V},$$

with *s*, *τ* = ±1, and

$$\begin{matrix}U & = & {{\rm{\Delta }}}^{2}+\frac{{J}_{{\rm{ex}}}^{2}}{2}+{\hslash }^{2}{v}_{F}^{2}{{\bf{k}}}^{2}+\hslash {v}_{{\rm{F}}}{J}_{{\rm{ex}}}{({\bf{m}}\times {\bf{k}})}_{z},\\ V & = & \frac{1}{2}\sqrt{{4{\rm{\Delta }}}^{{\rm{2}}}+{({J}_{{\rm{ex}}}+2\hslash {v}_{{\rm{F}}}{({\bf{m}}\times {\bf{k}})}_{z})}^{2}}.\end{matrix}$$

Schematic energy bands are depicted in Fig. 1(b), where the Dirac point is open in strong hybridization regime 2Δ > *J*_*ex*_, and it is closed otherwise. In the above, we have (2 × 2) energy bands represented by two indexes, *i*.*e*., *s* which has the same meaning as the spin index in the previous section, and *τ* which can be considered as the pseudo-spin index, which refers to the mixing of the top and bottom surface states. By using the same framework as in previous section, we can derive the torque field for each of the energy band as:

12 $${{\bf{H}}}_{s\tau }^{{\rm{eff}}}=s\frac{{J}_{{\rm{ex}}}}{\sqrt{{J}_{{\rm{ex}}}^{2}+4{{\rm{\Delta }}}^{2}}}\frac{n\hslash {v}_{{\rm{F}}}}{{\mu }_{0}{M}_{s}}(\hat{z}\times {\bf{k}})$$

for the strong exchange limit (${J}_{{\rm{ex}}}\gg \hslash {v}_{{\rm{F}}}{k}_{{\rm{F}}}$).

If the Fermi energy *E*_*F*_ = 0, only the two lower bands contribute to the torque field in Eq. (12), *i*.*e*., *s* = −1 and *τ* = ±1. Interestingly, the torque field in Eq. (12) does not depend on the band index *τ*. Therefore, the total torque field is given by ${{\bf{H}}}^{{\rm{eff}}}={\sum }_{s,\tau }{{\bf{H}}}_{s\tau }^{{\rm{eff}}}{f}_{s\tau }$, with *f*_*sτ*_ being the distribution function for each energy band. The total torque field is given as

13 $${{\bf{H}}}^{{\rm{eff}}}=\eta (\hat{z}\times {{\bf{j}}}_{e}^{ss}),$$

where

14 $$\eta =\frac{{\eta }_{0}}{\sqrt{1+4{({\rm{\Delta }}/{J}_{{\rm{ex}}})}^{2}}}.$$

**Figure 2:**
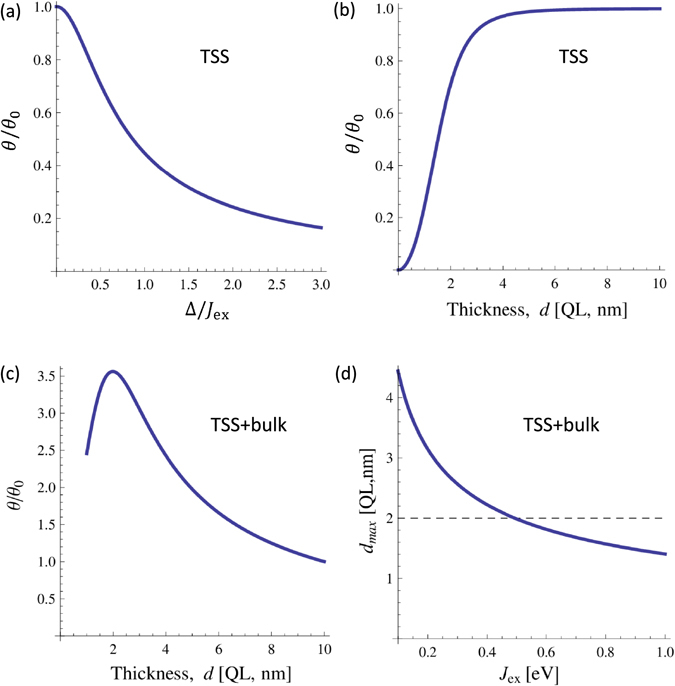
(**a**,**b**) Spin torque efficiency induced by topological surface states (TSS) in an ideal TI in the absence of the bulk channel. (**a**) Spin torque as a function of the tunneling coupling Δ. (**b**) Spin torque as a function of thickness *d*, *J*_ex_ = 0.2 eV. (**c**,**d**) Spin torque efficiency induced by TSS in the presence of the bulk channel. (**c**) Torque efficiency as a function of thickness. Comparing to the efficiency in thick film limit (*θ*_0_ normalized to 1), the maximum efficiency is about 3.5 times larger at an optimal thickness ${d}_{{\rm{m}}{\rm{a}}{\rm{x}}}\approx 3{\rm{n}}{\rm{m}}$. Parameters used: *J*_ex_ = 0.2eV, ${G}_{{\rm{TI}}}[{e}^{2}/h]\sim 19\,d$. (**d**) The value of optimal thickness as a function of exchange coupling for various TI parameter *B*_1_. The horizontal dashed line represents the effective thickness of the TI surface states (~2 nm). The peak of torque efficiency is only observed if the value of *d*_max_ is above the dashed line. *θ*_0_ is the efficiency at the limit of thick TI film. Parameters used: *B*_1_ = 0.1eV nm^2^ ^21^.

We see that the direction of the torque field in this case is still the same as in Eq. (6), *i*.*e*., in the transverse direction with respect to the applied current. However, the amplitude is now modified by a factor which is a function of the tunneling element Δ as in Eq. 14. Figure 2(a,b) depict the dependence of the torque efficiency $|{\mathscr{T}}|/{j}_{e}^{ss}$ on the tunneling element and the TI thickness, respectively. As expected, for a given surface state current *j* ^*ss*^, the torque efficiency is reduced when the Δ increases, a trend which can also be discerned from Eq. (12).

#### Thickness optimization

As discussed in previous sections, decreasing TI thickness gives rise to two opposite effects on the spin torque: torque reduction due to the strong surface hybridization, and torque enhancement due to the weak bulk channel. In this section, we will thus derive the optimal thickness to achieve maximized spin torque.

As the thickness of TI film is reduced, the conductance of the TI film is also diminished^18^. In extremely thin TI films, where the thickness is below 10 nm, the conductance is function of the thickness ${G}_{{\rm{TI}}}(d)={a}_{G}+{b}_{G}d$ [in units of *e* ^2^/*h*]. Meanwhile, in the thick-film limit, the conductance approaches an almost constant value^18^. Experimentally, ${b}_{G}\approx 19\,{{\rm{nm}}}^{-1}$ for Bi_2_Se_3_ thin films^18^.

The spin torque efficiency, which is the ratio between spin torque and charge current flowing in the TI, *i*.*e*., $\theta ={\mathscr{T}}/{j}_{{\rm{TI}}}$, is then

15 $$\theta =\mathop{\underbrace{\frac{{\eta }_{0}}{\sqrt{1+4{({B}_{1}{\pi }^{2}/{d}^{2}{J}_{{\rm{e}}x})}^{2}}}}}\limits_{{f}_{1}(d)\,increases\,with\,d}\mathop{\underbrace{(\frac{{G}_{{\rm{TI}}}^{ss}}{{a}_{G}+{b}_{G}d})}}\limits_{{f}_{2}(d)\,decreases\,with\,d}$$

The above expression is the product of two functions *f*_1_(*d*) · *f*_2_(*d*), where *f*_1_ (*f*_2_) increases (decreases) with *d*. In the limit of large *d*, ${f}_{1}(d)\sim {\eta }_{0}$, and *f*_2_(*d*) is relatively constant with the change in *d* as the conductance of the TI film is almost unchanged^18^, which means that the torque efficiency at large TI thickness would be almost constant. However, in the range of small *d*, the torque efficiency is more strongly dependent on *d*. In Fig. 2(c), the torque efficiency first increases as the thickness is reduced. This is due to the reduction of the bulk contribution to the TI conductance, which enhances the spin-polarized current from the surface state channel. As the thickness is further reduced, the torque efficiency becomes diminished as the hybridization effect becomes the dominant factor. Thus, the torque efficiency reaches a maximum peak at some optimal value of thickness *d*_max_.

Now we focus on the maximum efficiency and the corresponding thickness. Basically, these two quantities depend on system’s parameters such as *B*_1_, *J*_ex_. For simplicity, we consider the case where ${G}_{{\rm{TI}}}(d)={b}_{G}d$, for which the maximum efficiency can be analytically found as

16 $${\theta }_{{\rm{\max }}}=\frac{{\eta }_{0}}{\sqrt{4{\pi }^{2}{B}_{1}/{J}_{{\rm{ex}}}}}(\frac{{G}_{{\rm{TI}}}^{ss}}{{b}_{G}}),$$

corresponding to the optimal thickness of

17 $${d}_{{\rm{\max }}}=\sqrt{\frac{2{\pi }^{2}{B}_{1}}{{J}_{{\rm{ex}}}}}.$$

Taking value of the tunneling parameter *B*_1_ = 0.1eV nm^2^, and the exchange coupling *J*_ex_ = 0.1–0.5eV, the optimal thickness is estimated to be *d*_max_ = 2–5nm. In previous experiments^10–12^, the TI films have thickness in the range of 6–20nm, which is far above the predicted optimal values. We note here that, for the maximum torque to be observed, the optimal thickness should not be below the effective thickness of the TI surface states, *i*.*e*., *d*_max_ > 2nm^17, 18^ (see Fig. 2(d)).

## Discussion

One can check the underlying physics of the torque field reduction with the increasing surface hybridization in Eqs (13) and (14) by assuming the two surface states have the same helicity. By replacing $-{ {\mathcal H} }_{{\rm{TI}}}$ by ${ {\mathcal H} }_{{\rm{TI}}}$ in the bottom right block of Eq. (9), one can find the torque field is ${{\bf{H}}}^{{\rm{eff}}}={\eta }_{0}(\hat{z}\times {\bf{k}})$, which is independent of the tunneling coupling Δ. Therefore, we can conclude that it is the coupling between surface states with opposite helicities which reduces the net torque.

As mentioned earlier, we have assumed that the FM layer is only coupled to the top surface states, whereas its coupling with the bottom surface is ignored. Here, we will discuss how the torque is modified if the itinerant electron on the bottom surface may also couple to the FM, but with a weaker exchange coupling ${J}_{ex}^{^{\prime} }=\lambda {J}_{ex}$, *i*.*e*., with *λ* ≤ 1. In this case, the Hamiltonian of the bottom surface state becomes $-{ {\mathcal H} }_{{\rm{TI}}}+\lambda {J}_{{\rm{ex}}}{\bf{m}}\cdot \hat{\sigma }$. The torque field is read as ${{\bf{H}}}^{{\rm{eff}}}={\eta }_{\lambda }(\hat{z}\times {{\bf{j}}}_{e}^{ss})$, where

18 $${\eta }_{\lambda }=\frac{{\eta }_{0}(1-\lambda )}{\sqrt{{(1-\lambda )}^{2}+4{({\rm{\Delta }}/{{\rm{J}}}_{{\rm{ex}}})}^{2}}}.$$

It is easy to see that the coupling between the bottom surface and the ferromagnet leads to a reduction in the total spin torque, and it even vanishes for *λ* = 1. This results from the cancellation of the two surface states with opposite helicities. The equal exchange coupling is only possible if the film is extremely thin. In practice, this would not be the case since the film thickness should not be smaller than the effective thickness of the TSS (2 nm).

### Weak exchange limit

In the previous section, we have evaluated the spin torque and the thickness optimization in the strong exchange limit. Similarly, we can estimate the optimal thickness in the weak exchange limit, ${J}_{{\rm{ex}}}\ll \hslash {v}_{{\rm{F}}}{k}_{{\rm{F}}}$. In this case, the torque field is derived as

19 $${{\bf{H}}}_{s\tau }^{{\rm{eff}}}=s\frac{{J}_{{\rm{ex}}}}{\sqrt{4{\hslash }^{2}{v}_{{\rm{F}}}^{2}{k}_{{\rm{F}}}^{2}+4{{\rm{\Delta }}}^{2}}}\frac{n\hslash {v}_{{\rm{F}}}}{{\mu }_{0}{M}_{s}}(\hat{z}\times {\bf{k}})+s\tau \frac{\hslash {v}_{{\rm{F}}}{J}_{{\rm{ex}}}{({\bf{m}}\times {\bf{k}})}_{z}}{\mathrm{2(}{\hslash }^{2}{v}_{{\rm{F}}}^{2}{k}_{{\rm{F}}}^{2}+{{\rm{\Delta }}}^{2})}\frac{n\hslash {v}_{{\rm{F}}}}{{\mu }_{0}{M}_{s}}(\hat{z}\times {\bf{k}}).$$

Assuming the same distribution of the charge current in the system, the optimal thickness is calculated as ${d}_{{\rm{\max }}}=\sqrt{{\pi }^{2}{B}_{1}/\hslash {v}_{{\rm{F}}}{k}_{{\rm{F}}}}$. With the Fermi momentum *k*_*F*_ = 0.07–0.14Å^−1^ ^18, 26^, and the Fermi velocity *v*_*F*_ ~ 5 × 10^5^ m/s^21^, the optimal thickness is estimated to be in the range of ~1.4–2 nm. This range is almost below the effective thickness of the TI surface state, and thus the optimal spin torque may not be observed in the weak exchange regime.

## Conclusion

In this paper, we have analytically derived the spin-orbit torque induced by a thin TI film. First, we showed that in thin TI films, the spin torque is reduced due to the surface hybridization effect. This decrease is a consequence of the opposite helicities of the surface states on the two surfaces. On the other hand, lowering the thickness of the TI film may have a positive effect on the spin torque by reducing the bulk channel contribution to the total conductance. This increases the proportion of current flowing through the surface states, which constitutes the source of the current-induced torque on the magnetic layer. Due to these opposing trends, there thus exists an optimal TI thickness at which the torque efficiency is maximized. Based on typical experimental parameter values, we found the torque efficiency at the optimal thickness to be several times larger than the torque values at the thick TI-layer limit. Our prediction for the optimal thickness is within the practical range which can be verified experimentally.
